# Relationship between learning styles and interpersonal communication skills of nursing student in Medical Sciences Tehran University in 2012

**Published:** 2015

**Authors:** S Azari, S Mokhtari, H Mousavi, M Mohammadi, A Aliyari, M Salimi, GH Azari

**Affiliations:** * Health Management and Economics Research Center, Iran University of Medical Sciences, Tehran, Iran,; ** Health Economics, School of Public Health, Tehran University of Medical Sciences, Teheran, Iran; *** The Headquarters Students of Witness and Gallantry, Tehran University of Medical Sciences, Tehran, Iran,; **** Health Services Management, Ahvaz Jundishapur University of Medical Sciences, Ahvaz, Iran,; ***** Student Research Committee, Kermanshah University of Medical Sciences, Kermanshah, Iran,; ****** Student Research Committee, Kermanshah University of Medical Sciences, Kermanshah, Iran,; ******* Health Services Management Research Center, Institute for Futures Studies in Health, Kerman University of Medical Sciences, Kerman, Iran,; ******** Health Services Management, Department of Health Service Management, Islamic Azad University-Science and Research Branch, Tehran, Iran

**Keywords:** interpersonal communication skills, learning styles, nursing students, Medical Sciences Tehran University

## Abstract

**Introduction:** interpersonal communication skills are required for training and represent one of the most significant parts concerning the character of student learning. In another idea, learning is a constant method and learners favor a position of knowledge forms according to their character and individual practices. Evaluate the correlation between the learning methods and interpersonal conversation abilities of the nursing undergraduate in Medical Sciences Tehran University in 2012 was the purpose of this research.

**Methods:** In this regular detailed cross-sectional analysis, 361 students from the School of Nursing and Midwifery were chosen during a census method. The information collection instruments were regulated, giving a questionnaire called Interpersonal Communication Skills Standards exam and VARK Learning Styles questionnaire. Data was examined by SPSS application (18th edition) by using Mann-Whitney and Kruskal-Wallis test.

**Results:** 320 questionnaires were finished. 60.6% of the members were females. The average number of the students’ conversation abilities level was 101.91 ± 10.35. More than half of the samples (58.8%) preferred multi-modal learning styles (Bi-Tri and Quad Modals) and 41.2% of the students preferred single modal learning styles. There were no significant differences between the Interpersonal Communication Skills and the learning styles (P= 0.46).

**Conclusion:** According to no significant relationship between the communication skills of students with learning style and Demographic changeable and Lack of proper form of communication skills, we were ready to build different systems and courses related to improving the skills’ level.

## Introduction

The process of transferring the idea, message, information, and attitudes that provide the possibility of turning these data into action is called communication, which is considered one of the mankind’s basic and essential skills and like so many other skills, some have greater talent in this region [**1**]. Communication in medical science is a planned or programmed conversation aiming at presenting information to the patient, consulting, treatment, determining and solving problems [**2**]. In fact, communication is the means of exchanging information, concepts, values, and beliefs between individuals. Consequently, several categorizing methods have described communicative skills. In a group of cognitive, conceptual and process, abilities were introduced as basic and an essential part of communicative capacity and the different group split communicative talents into couple groups of central and excellent abilities, where communicative interpersonal abilities were considered among the basic communicative skills [**3**]. Reaching an adequate and learned style and approaching a step of moving contact using getting the latent and actual communicative abilities is named interpersonal [**3**]. Interpersonal communication occurs during crowd communicate with each other. In each communication, leastwise two people should participate, hear and answer to each other by different ways and it results in sending and receiving a message to accomplish a specific goal [**4**,**5**]. Communication is so important in human life that some scholars consider it the basis of all human development, individual damages and mankind development and progresses [**6**]. 

We should consider not only scientific, academic and professional abilities of a student but also the influence of students’ communicative skills, and such an effect is required regarding finding the patients’ benefits and improving their knowledge [**7**]. Having a regular and healthy communication does not free the expert groups from the need of goal-based and skill-based education in advanced levels; also private study is required to gain such abilities. Among the important factors effecting the students’ learning and quality of this learning, an individual practice of communicative abilities and because communicative abilities are educable could be considered, and a private data could be performed to transfer concepts and information to the students in the most efficient way possible [**8**]. 

Building an actual and private relationship should be a piece of health and medical care sector employees’ characteristics, this being of such value as it has important consequences on the patients’ comfort, increasing clinical outcomes and improving the patient’s partnership [**9**,**10**]. Proper communication between the medical team and the patient increases the patient’s health and decreases the rate of charges; Also, research has revealed that a limited relationship and communication of health and medical care sector employees and cases points to a little comfort of cases [**12**, **13**]. 

Also, learning is a standardized method, and it happens based on various scenarios for different people. Individuals prefer a collection made of learning methods and methods due to their personal personality and experiences [**14**]. The learning technique is a way of reasoning, processing, and understanding the knowledge that people experience, and use it to learning and working difficulties [**15**]. A method that a person knows and holds data from it and as a consequence gets experience and abilities are called learning method [**16**]. 

As a learner, every student has his/ her personal and unique learning style, and, the education scholars believe that the learner has different learning styles [**17**, **18**]. Teaching methods and educational guidelines of each learning style have differences compared with the other learning styles too. Creating the proper condition for the students’ learning and, as a conclusion, qualitative and quantitative success of educational method is hinge on the administrators and school authorities (such as professors and speakers of universities) understanding and experience of the students’ learning methods [**19**].

Isolated studies have investigated the method of managing the nursing students’ learning means and the interpersonal communicative abilities level [**20**-**24**], but yet, there has not been a study to determine the association among the learning method and the communicative interpersonal abilities, and what kind of learning method is preferred by each student and what type of connection exists between the learning style and the rate of communicative skills. Therefore, the present research was undergone while aiming to determine the relation between the learning method and communicative interpersonal abilities of the third and the fourth year nursing students of Medical University of Tehran through the year 2012. 

## Method 

The present research is a descriptive–analytical one, which was done in a sectional or periodic manner on the third and fourth year Nursing students of Tehran Medical University. The research society was made up of third and fourth year nursing students of nursing and topology faculty of Tehran Medical University. They were set to participate in the investigation using non-accidental single sampling system and because of the goal that sample volume was close to total sample society. Also, all third and fourth-year students took part in this study in the form of census and students in their 1st and 2nd educational year did not take part in it. The reason for choosing the third and fourth year students was their far greater clinical experience due to the fact that they passed more traineeship units. Therefore, all the students of these two years, which included 361 individuals, entered the research in the form of the census. 

The students’ cognitive understanding was achieved by giving a note of content containing the name and explanations about the study before the operation was begun. Data were gathered using a questionnaire including three parts. The first part included the students’ demographic information (educational semester, gender, average grade and the past of participating in the communicative skills workshop). The 2nd section of the questionnaire included standard learning methods of Warok from the book How Do I Learn Best by Fleming [**25**]. This questionnaire was also used in studies of Hamouzadeh et al., Peiman et al., Bahadori et al. and Salmi et al. [**26**-**29**]. Hinge on this questionnaire, seeing, listening, reading/ writing and movement/ motion methods of the student were prepared. This questionnaire involved sixteen questions and each question placed the learner in a learning condition. The members picked the part that bests given the information about their play in that position in each issue. If one of the choices was not enough to explain their condition, they could choose more than one item in each question. The third section of the questionnaire further involved the Standard Interpersonal Communication Abilities examination. This survey covered thirty-four 5item questions, and its number was based on 34 to 170. Each question choices were presented as almost never, rarely, sometimes, almost always and mostly and it was scored based on Likret points from 1 to 5. This survey was applied for durability and acceptability in Peipan et al. study [**30**]. Information was investigated using detailed statistics system and Kruskul-Wallis and Mann-Whitney U exam which was extra fit to examine the proper condition of data used in the Cilmograph-Smirnoff test. 

## Results 

Totally, 320 third and fourth year nursing students from the Medical University of Tehran were studied (return rate = 88.64%). One-quarter of samples (25%) were 8th term students, more than one-third (39.4%) were male and less than one-fifth (18%) had an average grade higher than 16. Only 57 individuals (17.8%) had the past of participating in communicative abilities workshops (**Table 1**). 

**Table 1:** Repetition of learners in the research based on term, gender, average grade and participating in communicative skills workshop

variant	Variant type	Variant (percentage)
Educational semester	Semester 8	80(25%)
	Semester 7	72(5/22%)
	Semester 6	76(8/23%)
	Semester 5	92(8/28%)
Gender	Male	126(4/39%)
	Female	194(6/60%)
Grade average	Less than 15	78(4/24%)
	Between 15-16	182(9/56%)
	More than 16	60(8/18%)
Workshop	Yes	57(8/17%)
	No	263(2/82%)

Based on Warok learning methods questionnaire, more than half of the samples (58.8%) used more one style of learning and 42.2% used only one style of learning. More than one-third of them (38.1%) used four models of learning concurrently (**Table 2**). 

**Table 2:** Repetition of learners under investigation based on using Warok learning methods

Warok learning style	Redundancy (percentage)
visual	34(6/10%)
listening	16(5%)
Reading and writing	25(8/7%)
performance	57(8/17%)
Two dimensional	29(1/9%)
Three dimensional	37(6/11%)
Four dimensional	122(1/38%)

Median and standard deviation of understudy students’ communicative abilities test’s score was equal to 35/ 10 ± 91/ 101. Based on Cilmograph-Smirnoff test, communicative skills test’s score did not have a normal distribution. Also, to define the relation between the communicative skills test’s score with the another demanding variants, non-parametric tests were applied. The minimum and maximum communication skills test score respectively belong to students who used reading, writing, and two-dimensional learning style. **Table 3** presents the median of communicative abilities score divided using Warok learning methods. As it can be observed, Kruskul-Wallis did not reveal a significant disagreement among the communicative abilities test score and Warok learning methods (p=0.46).

**Table 3:** The relation between using Warok learning methods and communicative interpersonal abilities of students

Warok learning style	Median and Standard deviation	P value
visual	8/10±8/100	46/0
listening	7/8±5/102	
Reading and writing	6/13±9/98	
performance	2/10±0/103	
Two dimension	0/9±6/103	
Three dimension	4/10±4/103	
Four dimension	9/9±3/101	

The maximum and minimum score of communicative abilities test belong, respectively to nursing students of semesters 5 and 6 where there was not a significant difference between the academic semester and the score of communicative skills test score (P=0.74). The median of male and female students grade did not differ much and the difference between their grade was not significant based on Man Withey test (P=0.79). The media of communicative abilities test score in students whose average grade was between 15 and 16 was less than the one of the other students, while students with an average grade higher than 16, had the highest score in their communicative skills, compared to the students who took part in communicative skills workshops (P=0.27). 

**Table 4:** The relation between semester, gender, average grade and participating in communicative skills workshop and communicative skills test score

P value	Median and Standard deviation		variant
74/0	92/10±32/102	Semester 8	Academic Semester
	86/10±65/101	Semester 7	
	29/11±28/103	Semester 6	
	44/8±62/100	Semester 5	
79/0	71/10±79/101	Male	Gender
	13/10±98/101	Female	
27/0	53/10±13/102	Less than 15	Grade average
	38/10±21/101	Between 15-16	
	92/9±73/103	More than 16	
27/0	29/11±39/103	Yes	Workshop
	12/10±59/101	No	

## Discussion 

The present research was realized while aiming to determine the relationship between the learning methods of the 3rd and 4th year nursing learners of the Medical University of Tehran with communicative interpersonal abilities during 2012. 

41.2% of the students used one type of style learning and 38.1% used four styles simultaneously. The mean and regular variation of communicative skills test of students under study was equal to 35/ 10 ± 91/ 101 which did not show a significant difference regarding the type of students’ learning style. Meanwhile, students with two-dimensional and four-dimensional style learning had a higher level of communicative skills in comparison with the individuals with one-dimensional style. What should more be considered is that the students’ communicative skills score was less than the average obtainable score of the questionnaire (102) which was not considered to be a satisfying score. 

Maslak Pak more noticed in investigating the communicative abilities of last year nursing learners that most of the students had a reasonable level of communicative skills [**24**]. The other outcomes of the investigation revealed that nurses and other medical employees were weak in creating a connection and communicating with patients [**30**-**33**]. Many types of research and studies reported that nurses have great problems in creating a communication with their patients [**34**, **35**] which were related to the outcomes achieved by the current study. Therefore, the essentiality to establish a proper communication should be maintained as a critical element of nursing services. 

No important variation was recognized among the academic semester and the communicative skills test’s score. However, students in all semesters had higher scores in relation with the first semester students. In research supported by Salimi et al., a significant relation was recognized between the students’ academic year and their level of communicative skills in a way that the third year students had the highest level of communication and the first year students had the deepest level [**3**]. Therefore, we could say that as the knowledge and experience of the students increased as a result of the theory and practical classes, their ability level and skill to create connection and communication went higher. 

Man-Whitney test did not reveal a striking contrast among the students’ gender and their communicative skill ability. Also, Hemmaty’s research did not disclose a significant relationship between the nurses’ speech communication performance of particular section and their gender [**36**]. At the same time, a study by Molaiee et al. on students of Ardabil Medical University concluded that the level of desired communicative skills among male and female students was statistically significant and female had a higher level [**37**]. The reason for such a difference could be related to the student’s study branch, because in Molaiee study, against studies of Hematy et al. and present research, only nursing students were included and various branches’ students took part. 

There was no significant relation between the average grade and the communicative skills test’s score of the learners, but the median of the communicative skills test’s score in students with a mean grade higher than 16, was more than one of the learners with a lower average grade [**3**], it was also recognized that as the students’ average grade increased, their level of interpersonal communicative skills also increased, but this increase was necessary to the scientific viewpoint. Despite the lack of a significant relation between the average grade and the communicative skills score, we could say that students who had a higher level of communicative interpersonal abilities during their education or academic period, also had a better academic progress condition. 

Communicative skills test’s score in students who participated in communicative abilities workshops was higher than that of students who did not; still Man-Whitney test did not reveal any important distinction. Khalifezade et al. research showed that the design of clinical education courses based on clinical monitoring and guideline pattern could improve interpersonal, professional, and communicative abilities of nursing students [**38**]. Therefore, considering that clinical education has a critical role in forming the professional abilities of nursing students, it is essential to have such courses. 

Taking into account the researchers of the present article, there has not been any similar research or study about the connection among the learning methods and communicative interpersonal abilities of nursing students until now. Therefore, it is advisable and suggested to present the researches and studies that consider these two factors to other branches and graduates in the working environment. 

## Conclusion 

The level of students' communicative abilities is independent of their learning method and also to their information about it. Also, the nursing students’ achieved score did not have a satisfactory level. What should be taken into account is that in the recent year, the educating communicative skills have become the role of the advanced countries nursing education program and meanwhile no special plan has been set for teaching communicative skills to medical students during the clinical education in our country. Therefore, to correct the existing condition, the level of communicative skills among the nursing learners and other medical sciences students should be increased. The level of these skills could be improved by creating a new educational plan for the mentioned students in relation to nurses’ communicative abilities and also presenting related courses. 
